# Harnessing Genomics and Transcriptomics Approaches to Improve Female Fertility in Beef Cattle—A Review

**DOI:** 10.3390/ani13203284

**Published:** 2023-10-21

**Authors:** Nicholas C. Kertz, Priyanka Banerjee, Paul W. Dyce, Wellison J. S. Diniz

**Affiliations:** Department of Animal Sciences, Auburn University, Auburn, AL 36849, USA

**Keywords:** beef heifer selection, fertility, genomics, transcriptomics, systems genomics

## Abstract

**Simple Summary:**

Cow–calf production is the foundation of the U.S. beef industry. However, infertility and sub-fertility limit its sustainability. In this review, we discuss the progress made as a result of the implementation of genomics and transcriptomics technologies to understand the genetic basis underlying fertility-related traits in cattle. We highlight the opportunities and limitations of using such technologies to discover genes and regulatory mechanisms and discuss the importance of translating research findings from bench to on-farm applications.

**Abstract:**

Female fertility is the foundation of the cow–calf industry, impacting both efficiency and profitability. Reproductive failure is the primary reason why beef cows are sold in the U.S. and the cause of an estimated annual gross loss of USD 2.8 billion. In this review, we discuss the status of the genomics, transcriptomics, and systems genomics approaches currently applied to female fertility and the tools available to cow–calf producers to maximize genetic progress. We highlight the opportunities and limitations associated with using genomic and transcriptomic approaches to discover genes and regulatory mechanisms related to beef fertility. Considering the complex nature of fertility, significant advances in precision breeding will rely on holistic, multidisciplinary approaches to further advance our ability to understand, predict, and improve reproductive performance. While these technologies have advanced our knowledge, the next step is to translate research findings from bench to on-farm applications.

## 1. Introduction

The livestock industry is a critical component of the agricultural sector in the United States (U.S.). In 2022, cattle production was anticipated to represent about 17% of the USD 462 billion in total cash receipts for agricultural commodities [1]. Despite cattle numbers declining since 1975, beef production has increased due to advances in management practices, genetics, and nutrition. The beef industry needs to address global issues such as climate change and the use of natural resources in a sustainable manner while producing more food to feed a growing population [2]. Consequently, the future of a sustainable beef industry relies on improved reproductive performance and growth efficiencies. Fortunately, the cow–calf sector holds immense potential (reviewed by [3,4]) to positively contribute towards addressing those emerging issues [5,6].

Cow–calf production is the foundation of the U.S. beef industry, representing 86% of beef operations and 84% of the beef cattle population in the country [7]. However, reproductive inefficiency has been a significant cause of economic loss and a limiting factor for the sustainability of the beef industry [8,9,10]. The annual gross loss (due to pregnancy failure) of beef females is estimated to cost USD 2.8 billion in the United States [10]. Strategies to mitigate such losses include selecting and developing heifers with high genetic merit with respect to fertility traits. In addition to being a source of genetic gain for the herd, replacement heifers also represent a large portion of the capital outlay invested by producers [11,12]. Consequently, the selection of heifers is critical for the overall profitability and sustainability of the cow–calf sector. Cushman et al. [13] reported that heifers that calved earlier in the breeding season remained longer in the herd and weaned more kilograms of calf throughout their productive life. 

Despite the consensus that good fertility and longevity are essential for the sustainability of the cattle industry, improving reproductive efficiency has been a major challenge worldwide [14,15] due to the various complex factors underlying fertility and related traits. Indicator traits of fertility tend to be low in terms of heritability, are expressed late in life, and are controlled by many genes of small additive and non-additive effects [16,17,18,19]. Still, there is enough genetic variation underlying fertility to support the improvement of reproductive efficiency [14,17,18]. The complex nature of fertility has necessitated the integration of disciplines such as reproductive biology and genetics to accelerate genetic gain [20]. Likewise, the advent of “omics” technologies has provided new capabilities to analyze the structure and function of an organism at different regulatory levels, including DNA (genomic), RNA (transcriptomic), proteins (proteomic), and metabolites (metabolomic) [21]. These advances have improved our understanding of the genetic architecture governing fertility and have allowed for the more accurate selection of animals with high genetic values [20,22].

Genomic selection (GS) has contributed to the increased productivity of yield traits and the genetic gain of low-heritability traits such as fertility, especially in dairy cattle [23,24]. Although promising, findings from different research groups around the globe are still limited, as no major genes or genetic variants regulating fertility have been reported. Other omics-based approaches have increasingly been used to dissect the molecular basis of fertility and provide a more comprehensive understanding of the biological pathways associated with reproductive success [25,26,27,28,29]. Advances in metabolomics and proteomics applications in relation to cow fertility were discussed by Aranciaga et al. [30]. Hence, in this review, we discuss the status of the genomics and transcriptomics technologies currently applied to female fertility and the tools available to cow–calf producers to maximize genetic progress. While our focus herein is on beef cattle, we will occasionally draw a parallel with results regarding dairy cattle to fill the current gaps in the literature. We did not intend for this paper to be an exhaustive review; instead, our aim was to describe the recent findings and advancements regarding the use of genomics, transcriptomics, and systems genomics analyses, especially with respect to beef fertility. Furthermore, we will discuss the opportunities and current limitations of using these approaches to discover genes and regulatory mechanisms. Opportunities to translate research findings from bench to on-farm applications are also discussed.

## 2. Female Fertility: From Biology to Economics

Fertility is a general term encompassing a variety of traits that contribute to overall reproductive success [31]. Reproductive or fertility-related traits have been recorded in several ways, and authors have proposed different measurements. Berry et al. [17] classified female reproductive traits into three categories: interval (e.g., calving interval), binary (e.g., pregnant or non-pregnant), and count (e.g., number of services) traits. Additional reproductive measurements and definitions have been reviewed by Berry et al. [17], Cammack et al. [19], and Kgari et al. [32]. Although the relative importance of such measurements will differ between production systems and breeding objectives, fertility is critical for the profitability of cattle operations [17,33]. Herein, we will broadly consider fertility to be defined as “the ability to conceive, maintain a pregnancy, and deliver a normal, living calf” [20,33].

Historically, selection has focused on the increased performance and growth of beef animals [15]. Especially in dairy cattle, producers’ priorities were related to milk production due to its direct effects on profitability [32,34]. Nonetheless, over the years, the cattle industry has reported declining reproductive success due to the inverse relationship between production and fertility traits [17,18,34]. Thus, due to these unfavorable correlations, several breeding programs are now including fertility traits in their genetic evaluations [31]. 

### 2.1. Heritability and The Traditional Selection of Fertility Traits

Fertility is a complex, quantitative trait determined by a large number of variants with small effects that are spread throughout the genome [31,35]. In general, indicator traits of fertility are often low in terms of heritability, with values ranging from 0.02 to 0.1 [17,19]. However, the heritability of interval traits tends to be greater than the values from binary or count traits [17]. Heritability provides a measurement of genetic variation and is an important parameter in the prediction of genetic gain, as it expresses the reliability of the phenotypic value as an indicator of the breeding value [36]. Heritability can be defined as the proportion of phenotypic variation due to genetic differences among individuals. Many contributing factors are related to the low heritability of fertility traits, including the environment and the number of records available [18]. Management and nutritional programs affect various aspects of fertility, such as age at puberty, age at breeding, reproductive development, and longevity [11]. Therefore, an increased, accurate number of records and a uniform environment are important to improve the accuracy of heritability estimates and accelerate genetic gain [37]. Additionally, as will be discussed later, genomic selection has improved the accuracy of selecting low-heritability traits [17].

Only recently, fertility traits were included in breeding programs. The selection of fertility traits in beef cattle has its own challenges due to the diversity of breeds, production systems, and breeding goals [37,38]. Additional challenges are related to the records from extensively managed herds and the availability of suitable data for genetic evaluation [37]. The female reproduction traits that have been used for genetic evaluation include heifer pregnancy rate, days to calving, age at first calving, calving interval, and stayability [37]. The Beef Improvement Federation (BIF) has provided guidelines for Uniform Beef Improvement Programs and the traits to be recorded for genetic prediction [39]. Likewise, the National Cattle Evaluation Consortium (NCEC) has measured economically important traits across the most prominent beef cattle breeds in the U.S., although the evaluation of reproductive traits is still limited [40]. A summary of reproductive traits for which Expected Progeny Differences (EPDs) are reported is shown in Table 1. 

The EPDs reported in Table 1 represent the direct genetic potential of sires to be transmitted to their offspring. Although limited by low accuracy, they allow producers to select sires that can improve reproductive performance [41]. Another opportunity for advances in beef cattle fertility is in the selection of replacement heifers. These animals are the genetic future of the cowherd and represent a large portion of the capital outlay invested in cow–calf production [11]. However, most producers still use phenotype-based strategies to select replacements for the breeding herd. As fertility-related traits have low heritability, phenotypes are poor predictors of an animal’s genetic value, leading to slow genetic progress. Most commonly, body weight, reproductive tract score, pelvic dimensions, body condition score, and structural correctness are the traits considered during the selection process [11]. Although age at breeding, growth, and reproductive maturity are key traits influenced by the timing of puberty, and thereby, fertility, they are not the most accurate predictors of reproductive success [8,42]. Hence, as will be discussed later in this review, researchers have proposed the development of novel traits that could better predict fertility potential [33,37,43]. 

### 2.2. Economic Impact of Fertility in Beef Cattle Production

Fertility and reproductive success are directly linked to the economics of cow–calf production operations [19,44,45]. Developing a calf into a heifer that calves out, on average, within two years of age requires a significant amount of a producer’s time and money. However, reproductive failure is the primary reason cows are sold in the U.S. According to the USDA, 43.9% of cows sold in 2017 for purposes other than breeding were due to reproductive failure, i.e., reproductive problems and pregnancy status (open or aborted) [46]. Additionally, 16.4% of all beef cows aged less than five years old were culled. Although this is a lower percentage, it represents a large amount of monetary loss for beef cattle producers, as a cow is expected to produce, on average, five to six consecutive calves in order for the producer to recoup development costs and begin to turn a profit [8,44,47]. 

Considering the percentages mentioned above, the selection of replacement heifers is critical to the sustainability and profitability of a cow–calf operation. Heifers are not only the genetic future of the herd but also, if the opportunity cost is factored in, they are the greatest investment beef producers can make [11,42]. Dickinson et al. [48] reported that, in their study, on average, 15% of heifers failed to become pregnant during their first breeding season. These authors showed that traditional traits used for heifer selection, such as age at breeding, body condition score, and reproductive tract score, alone were not predictive of reproductive outcomes [48]. On the other hand, Hindman et al. [42] reported that age at AI and pelvic width were associated with heifer pregnancy through AI. These conflicting results reinforce the need for further research to develop accurate selection strategies that allow for differentiation between fertile and infertile heifers at an early stage. 

## 3. New Opportunities for Old Challenges: Novel Traits and Technologies

Efforts have been made to identify the novel traits that need to be recorded for genetic evaluation as well as the identification and use of genetic markers through omics approaches. Novel traits such as age at calving, days to calving, first calving interval, and heifer pregnancy have been proposed for inclusion in breeding programs as they have, on average, higher heritability than previously used fertility-related traits [37,49]. The estimated heritability for the first calving interval was 0.23 in Charolais [50]. Likewise, the estimates of age at first calving, days to calving, and pregnancy from heifers in the Angus Australia database were 0.25, 0.26, and 0.32, respectively [49]. 

It is important to highlight that advances in omics technologies and analytical methods have provided opportunities to accelerate the genetic progress of low-heritability traits. These technologies can provide a snapshot of a vast amount of molecules in a tissue or cell [51]. The primary goal of such approaches is to provide a deeper understanding of animal biology and the genetic architecture and function that underlie a trait so that we can connect the animal genome to its phenome [52]. DNA-based technologies and the emergence of genomics have led to significant advances in identifying genetic variants affecting fertility [35]. Functional genomics approaches such as transcriptomics and proteomics have shed light on the regulatory mechanisms modulating reproductive processes. Likewise, analytical approaches to integrate different sources of omics data and model the interactions between molecular regulatory layers have provided insights into the interplay among fertility trait variation and the various levels of genome regulation. In the following sections, we provide an overview of the main findings on the genetic progress of female fertility traits based on genomics and transcriptomics approaches. Furthermore, we describe a few studies that have implemented systems genomics analysis. 

### 3.1. Genomics

The development of high-density genotyping assays based on SNPs (single-nucleotide polymorphisms) has led to major genetic progress in livestock species [52,53,54]. The dairy industry has made great advances with the implementation of GS to improve selection accuracy [52,53]. Wiggans et al. [53] and García-Ruiz et al. [55] reported on the dramatic changes in the dairy industry since the implementation of GS in 2008. They reported a reduction in the generation interval and increased genetic gain for low-heritability traits such as fertility. For example, genomic predictions for daughter pregnancy rate caused a gain of 17% in reliability [53]. On the other hand, the adoption of GS in the beef industry has been limited despite evidence of its benefits. Factors related to that include the large number of breeds and crossbreds, the limited extent of phenotyping, and the low number of offspring per female [38]. 

Lately, breed associations have partnered with biotechnology companies to develop genomic tools to support GS in beef cattle [41,49]. Likewise, breed associations are using genomic information in genetic evaluation programs to provide genomic estimated breeding values (or genomic-enhanced EPDs). Examples of such tests are presented in Table 2. To the best of our knowledge and at the time of writing this review, there are no peer-reviewed publications on the performance of such tests for the selection of heifer fertility traits. For production and carcass traits, Arisman et al. [56] reported that genomic predictions based on the GeneMax^®^ advantage test accurately predicted the heifer’s genetic merit. The Angus Australia Association, in partnership with Zoetis and CSIRO, also developed a genomic-based tool for replacement heifer selection (HeiferSELECT^™^) [49,57,58]. This test provides genomic-enhanced breeding values (GEBVs) for traits related to cow–calf production, feedlot performance, carcass quality, and resilience [58]. Based on the Angus Australia database, Alexandre et al. [49] assessed the potential to include fertility indicator traits in the Angus HeiferSELECT trait repertoire. The accuracies for age at first calving, days to calving, and pregnancy were 0.27, 0.24, and 0.34, respectively [49]. Additionally, heritability values ranged from 0.25 to 0.32. The potential for genomics to contribute to the genetic improvement of beef cattle fertility is evident from the findings reported above. However, further advances will be achieved as we increase the number of animals phenotyped and the array of traits recorded [37,40]. 

It is essential to highlight that genomics has improved the understanding of the genetic regions modulating fertility and identified candidate genes through genome-wide association studies (GWAS). These studies have revealed thousands of quantitative trait loci (QTLs) associated with economically important traits, including fertility. Table 3 shows a summary of selected GWAS studies in beef cattle. Although no genes with major effects have been reported so far, this knowledge has supported the development of strategies to improve reproductive function [54]. The CattleQTL database (CattleQTLdb, release 50) currently has 193,898 QTLs/associations from 1130 publications [63]. Considering reproduction-related associations only, the database reported 21,438 and 20,115 QTLs for “general reproduction parameters” and “fertility traits,” respectively [63]. Most studies, however, have focused on dairy breeds. Ma et al. [18] reviewed the main GWAS studies deposited in the CattleQTLdb addressing fertility in Holsteins. The authors highlighted that five out of the eight selected studies harbored a QTL in Chr 18 associated with fertility traits, including cow conception rate, daughter pregnancy rate, and heifer conception rate. 

As in dairy cattle, the GWAS studies in beef cattle have increased the accuracy of prediction for reproduction traits. Research on Brahman and Tropical Composite cattle from Australian herds showed that early reproductive performance traits had moderate to high heritabilities [37]. Based on single and multi-trait models, Olasege et al. [22] estimated the genetic parameters of six female fertility traits recorded early in life. Among them, age at detection of first corpus luteum (AGECL) had the highest estimates for both models in Brahman (single-trait: 0.56 ± 0.08; multi-trait: 0.57 ± 0.08) and Tropical Composite breeds (single-trait: 0.46 ± 0.08; multi-trait: 0.69 ± 0.02). Interestingly, blood concentrations of insulin-like growth factor 1 (IGF-1) measured in cows at 18 months were suggested as a predictor of female fertility [22]. Fortes et al. [64] examined variants associated with AGECL in the Tropical Composite breed and reported 2799 significant SNPs (P < 0.05). Among the candidate genes related to AGECL, the authors reported *NMDAR2B*, *SPOCK1,* and *ZNF462*. 

**Table 3:** Summary of selected genome-wide association studies for fertility-related traits in beef cattle.

Breed	Number of SNPs	Associated Traits	Number of Candidate Genes	Number of Samples	Reference
Crossbred	20	^a^ AFC, RTS	18	293	[65]
Crossbred	19	PBW, PR, ^b^ AFC	Not listed	785	[12]
Crossbred	1	TBRD	2	239	[66]
Red Angus	21	HPG, STAY	13	9776	[67]
Nelore	^c^ Top windows	HR, NC53	489	2925	[68]
Nelore	42	HR, AFC	35	2056	[69]
Nelore	^d^ Top windows	HP, NF	204	1267	[70]
Angus ^e^	2	F, SF	1	22	[29]
Crossbred	6	HF, SF, IF	10	36	[71]
Angus crossbred	28	HF, SF	19	85	[72]

^a^ AFC: Antral follicle count; ^b^ AFC: Age at first calving; ^c^ the authors reported the top 20 1-Mb significant windows for HR and NC53; ^d^ the authors reported genomic windows that explained more than 1% of the additive genetic variance; ^e^ the authors performed a GWAS for Holstein and Angus together; RTS: Reproductive tract score; PBW: Pre-breeding bodyweight; PR: Pregnancy rate; TBRD: Number of services required to successfully conceive and maintain pregnancy; HPG: Heifer pregnancy; STAY: Stayability; HR: Heifer rebreeding; NC53: Number of calves at 53 months of age; HP: Heifer pregnancy; NF: Number of antral follicles; F: Fertile; SF: Sub-fertile; HF: High-fertile; IF: Infertile; details on SNPs and candidate genes are reported in Supplementary Table S1.

**Figure 1:**
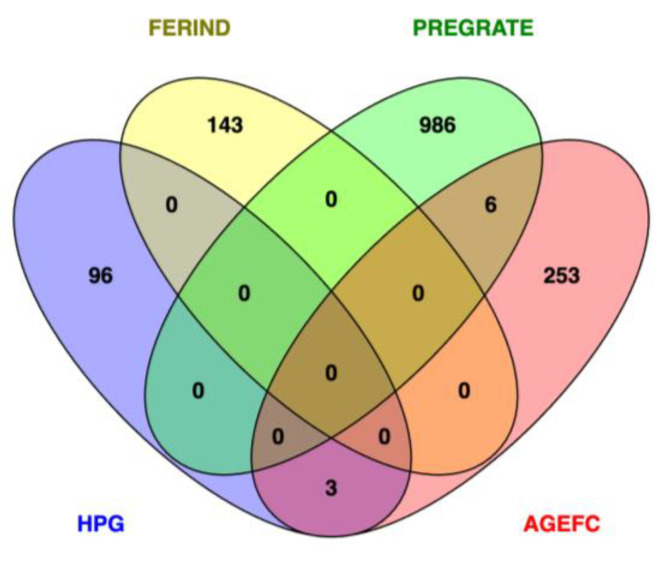
Overlapping of single-nucleotide polymorphisms associated with fertility-related traits from the CattleQTL database. FERIND: Fertility index; PREGRATE: Pregnancy index; AGEFC: Age at first conception; HPG: Heifer pregnancy.

Similar approaches have been used to investigate the genetic variants associated with age at first calving, heifer rebreeding, and stayability in Nelore cattle [68,69,73]. The genetic basis of novel phenotypes such as antral follicle count (AFC) and reproductive tract scores have also been investigated in crossbred beef heifers [65]. Stegemiller et al. [65] reported 14 and 6 significant SNPs associated with AFC and RTS, respectively, and reported that *BMP6, PLEHKM3, IDH1, MAP2,* and *FOXO6* were among the candidate genes. It is important to highlight that although these studies have provided a better understanding of the genomic regions governing fertility, they have not yet provided causal variants or major regulatory genes. Furthermore, there is little overlap in terms of the genes or markers mentioned across multiple studies. For example, we retrieved 1483 SNPs from the CattleQTLdb associated with four fertility-related traits. As shown in Figure 1, only six (rs110249413, rs110457668, rs110469756, rs110474527, rs42938737, rs43480825) SNPs were shared between pregnancy rate (PREGRATE) and age at first conception (AGEFC), and three (rs108940570, rs133503069, rs134601255) were shared between heifer pregnancy and AGEFC. The differences in the methods used for phenotyping or data analysis [74] and small sample sizes are reasons for the shared, limited QTLs or markers across multiple studies [18]. 

### 3.2. Transcriptomics 

As a step forward in dissecting the molecular basis of fertility, transcriptomic studies have become a powerful tool to understand how gene expression controls cellular functions and their role in determining specific phenotypes [75,76]. In measuring the genome-wide expression of RNA molecules such as mRNAs, miRNAs, and lncRNAs, the assumption is that differences in dynamically expressed RNAs can partially explain phenotypic variation across animals [76]. Divergence in gene expression may result from differences in the physiological status of the animal at a specific time point and genetic variation in regulatory regions [77]. Studies in humans and livestock species have focused on using transcriptomics approaches to identify useful biomarkers for predicting fertility. From an animal breeding standpoint, the discovery of functional mutations that are directly associated with cattle fertility will support the development of genetic markers that can be used in genetic improvement programs [52]. 

The number of transcriptomic studies is rapidly growing, as researchers have targeted different tissues, experimental models, and biological conditions to identify differences in the abundance of the genes that underlie fertility-related traits. Similarly, several tools and methods have been proposed for transcriptomics analysis (reviewed in [78,79]). New opportunities have been unveiled by searching for circulating RNA molecules that could provide information on the physiological status of reproduction-related organs [80,81,82]. Likewise, exosomes have been associated with fertility potential in cattle. Mitchell et al. [83] reported significant differences in the number of plasma exosomes between fertile and sub-fertile dairy cows. A series of studies have characterized the transcriptomic profile differences of the endometrium [84], corpus luteum [85], liver and muscle [86], and other tissues [21], especially in dairy cattle. Beerda et al. [77] reviewed the findings of transcriptomic studies underlying dairy fertility. Because of the extensive amount of publications on dairy cattle, we will mainly describe recent results regarding beef cattle.

Genetic merit for fertility traits affected puberty and age at pregnancy in heifers, leading to long-term effects on the performance of cows [13,87]. Fortes et al. [88] investigated the changes in the gene expression profile of the uterine tissue by comparing pre- and post-pubertal Brahman heifers. These authors reported 26 significantly differentially expressed genes and 100 transcription factors (TFs) as potential regulators of differential expression, including *SOX2*. This TF is involved with the reorganization of the endometrium during embryo attachment and implantation [72]. Neupane et al. [72] highlighted *SOX2* and *OCT4* TFs as the master regulators of 773 genes associated through a GWAS with fertility in beef cattle. Under a similar experimental design, Cánovas et al. [26] profiled the hypothalamus, pituitary gland, uterus, endometrium, ovary, adipose, liver, and *Longissimus dorsi* tissues from Brangus heifers. Among the differences in gene expression between pre- and post-pubertal heifers, the authors highlighted that most of the differentially expressed genes (DEGs) from the hypothalamus were upregulated in pubertal heifers. Additionally, the authors reported a coordinated regulation between the endocrine response of the hypothalamus and signals from the adipose tissue [26]. 

Multiple factors are involved with infertility or sub-fertility in cattle, including progesterone signaling and uterine receptivity [88,89]. Using serial embryo transfers (ET) to classify heifers as high-fertile, sub-fertile, and infertile, Geary et al. [90] reported major transcriptional changes in the endometrium at day 14 after ET. Also using ET, Martins et al. [91] investigated the effects of progesterone (P4) on modulating the gene expression of uterine luminal epithelial cells and its potential effects on the odds of pregnancy in *B. indicus* influenced recipient cows. The authors reported 240 P4-modulated genes that were involved in oxidative phosphorylation, biosynthetic activity, and the proliferation of epithelial cells. Furthermore, 292 genes affected by P4 negatively influenced the odds of pregnancy [91]. Based on the main findings, the authors concluded that a reduction in the activity of the innate immune system and signaling by phosphoinositide-dependent pathways were determinants of uterine receptivity [91]. Silva et al. [92] investigated the hormonal profile of crossbred cows and the effects of contrasting concentrations of P4 on the transcriptome profile of uterine luminal epithelial cells on days 4, 7, and 14 after estrus. Dynamic temporal changes were observed by examining the number of DEGs identified between the high- and low-P4 groups (518, 905, and 14,409 DEGs on days 4, 7, and 14, respectively) [92]. In alignment with the findings of Martins et al. [91], Silva et al. [92] reported pathways related to the activation and signaling of the innate immune system at day 4. The differences at day 7 were related to a downregulation of the network of the extracellular matrix remodeling process, followed by an exacerbated inflammatory response at day 14 [92]. 

Transcriptomic studies have been implemented to investigate the fetal–maternal crosstalk during the recognition of pregnancy and the interplay of P4 on conceptus elongation [93,94,95]. The review by Rabaglino [96] is a great resource for understanding the transcriptional landscape and the current findings derived from studying blastocyst and concept elongation in the context of cattle. Mamo et al. [93] reported conceptus-induced transcriptome changes in the pre- and peri-attachment periods. In this context, it has been shown that both P4 concentration and the presence of the conceptus alter the endometrial expression of secreted protein-coding genes (*PLIN2, TINAGL1, NPNT, LCAT, NMN,* and *APOA1*) [94]. Two other studies comparing the fertility status of beef heifers found that there were no differences in P4 concentrations in serum during the first 16 or 20 days post-estrus between pregnant or non-pregnant heifers [71,90]. Similarly, in [90], P4 levels were not different between the fertile, sub-fertile, or infertile groups. However, the endometrium transcriptome profile of these heifers differed between the groups on day 14 after embryo transfer. Based on their comparisons of their DEG findings, the authors suggested that infertility could be related to the differences in the innate and adaptive immune systems in the endometrium [90]. 

**Figure 2:**
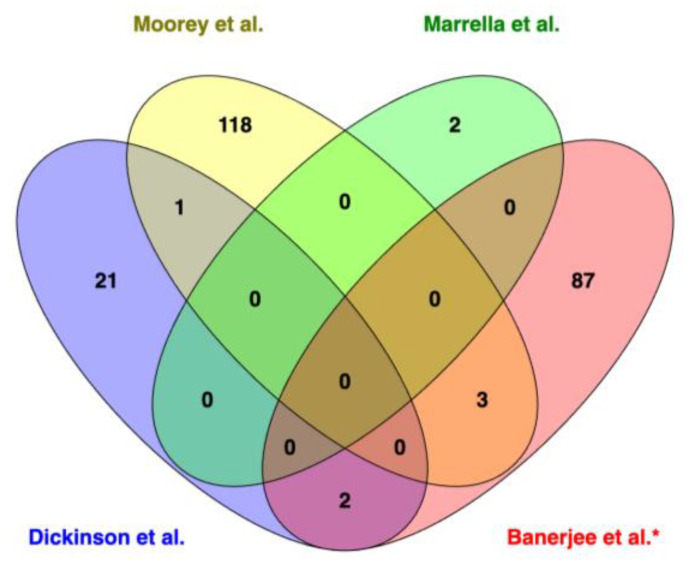
Overlapping of differentially expressed genes of peripheral white blood cells in beef heifers classified as pregnant or non-pregnant. Gene lists were retrieved from Dickinson et al. [97], Moorey et al. [82], Marrella and Biase [29], and Banerjee et al. [104]. * Transcriptome profile measured at weaning. Details on the differentially expressed genes across studies are reported in Supplementary Table S2.

Blood-based mRNA has been proposed as a potential avenue to identify biomarkers for early diagnosis of infertility [29,82,97] and to understand the fetal–maternal interaction during pregnancy recognition [98]. As previously mentioned, blood is an ideal surrogate material as it can reflect physiological changes in other tissues and organs [99,100]. Additionally, functioning as a mediator of the immune response [100], circulating transcripts in the blood can provide an overview of the immune system [98,99]. Significant differences were reported in the transcriptome profile of peripheral white blood cells (PWBCs) at the time of artificial insemination (AI) from heifers that became pregnant and those that were open at the end of the breeding season [82,97]. In their study, Dickinson et al. [97] reported that six DEGs were shared between pregnant and open Angus–Simmental heifers. In a similar experiment, Moorey et al. [82] reported 67 and 81 protein-coding DEGs between AI-pregnant vs. non-pregnant and natural breeding vs. non-pregnant heifers, respectively. Interestingly, *MNS1* is the only gene overlapping both studies (Figure 2). While no reports exist on the role of *MNS1* in female fertility, it has been shown that this gene is essential for spermiogenesis in mice [101] and testicular development and spermatogenesis in Simmental bulls [102]. However, both studies reported the immune-related pathways over-represented by the identified genes. Marrella and Biase [29] identified the DEGs *APMAP* and *DNAI7* in PWBC from Angus heifers at AI. However, the proteins encoded by these genes were not differentially abundant between fertile and sub-fertile heifers [29]. 

Further analysis using a co-expression framework on the data generated by Dickinson et al. [97] showed DEGs differentially connected between the AI-pregnant and non-pregnant groups [81]. For instance, *TAC3* was identified not only as a hub gene in the non-pregnant heifers but also as being more connected in the network. Hubs are central to the network’s topology and are likely essential for the associated trait [103]. The findings suggested a differential rewiring of key hub genes that may play a critical role in fertility [81]. We reported similar results when measuring the PWBC’s transcriptome profile at weaning of heifers retrospectively classified as fertile or sub-fertile [104]. In this study, we reported 92 DEGs, among which the key hub genes from sub-fertile heifers were over-represented for immune response and cytokine production pathways. 

We overlapped the DEGs identified in publications on the PWBC transcriptome, and only a few of them were shared (Figure 2). Like the GWAS results, there was limited overlapping of DEGs across studies. Gene expression is strongly affected by environmental factors. Likewise, breed composition, reproductive management, and analytical and methodological differences make it challenging to compare the results. Interestingly, five DEGs identified at weaning [81] were found by Dickinson et al. [97] (*LYZ* and *TFF2*) and Moorey et al. [82] (*CSF3R*, *NLRP1,* and *ENSBTAG00000049346*) at breeding (either through AI or natural breeding). However, only *TFF2* displayed the same direction of expression (upregulated in non-pregnant heifers). Over-represented immune-related pathways were reported in all the studies [81,82,97,104]. Collectively, these results suggest that changes in immune modulation at weaning carry on into breeding, likely affecting pregnancy outcomes. Moreover, changes in immune pathways were shared between blood and uterine tissues [81,82,90,91,92,97,104]. Based on these findings, it is critical to conduct further research on the crosstalk between immune and reproductive function. Understanding their interaction and potential mechanisms may support strategies to improve fertility through immune modulation. 

These findings hold promise for advancing our knowledge of the regulatory mechanisms associated with gene regulation. However, due to many layers modulating gene expression, it is challenging to identify consistent and reproducible biomarkers. Ross et al. [105] investigated 13 fertility-related genes from eight tissues in Brahman cows using three different sequencing technologies. They reported extensive variation across genes and tissues and highlighted the power of complementary approaches to characterize complex traits. In this context, metabolomics and proteomics are emerging approaches for predicting fertility. The profiling of follicular fluid and blood serum/plasma has been used to investigate female fertility [106,107]. In-depth reviews of these technologies and their applications in cow fertility have been conducted by Aranciaga et al. [30] and Fair [108]. In addition to the opportunities provided by individual analysis, it has been shown multi-omics approaches improve our ability to fully harness the interaction between regulatory layers.

### 3.3. Integrating Genomics to Transcriptomics

Genomics and transcriptomics approaches have been successful in identifying genetic variants and genes to untangle the genetic architecture of fertility-related traits. However, most of the associated variants are harbored in non-coding or intergenic regions [109]. This suggests that genetic variants may affect fertility via the differential modulation of gene expression [35,110]. Thus, integrating genomics with transcriptomics can shed light on the interplay between genotype and fertility [52]. Such an approach is known as systems genomics, which allows for the identification of functional variants called expression quantitative trait loci (eQTLs) [111]. eQTLs are regions harboring SNPs that can modulate or control genome-wide gene expression [111,112]. They can affect nearby genes (*cis*-eQT, < 1 Mb) or genes in a different locus or chromosome (*trans*-eQTL, > 1 Mb) [110,111,112]. 

**Figure 3:**
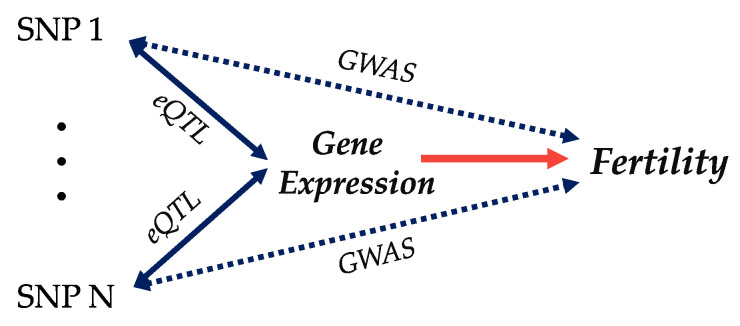
Genome–transcriptome–phenotype data integration via systems genomics to untangle the regulatory mechanisms involved with fertility-related traits. Reprinted from He et al. [113] with permission from Elsevier.

Figure 3 shows that GWAS analysis can directly associate SNPs with the phenotype. Likewise, the differences in gene expression between fertile and infertile animals can help pinpoint candidate biomarkers. However, under a systems genomics approach, eQTLs from the direct association between SNPs with gene expression can help prioritize causal variants and reveal the precise biological mechanisms through which DNA differences influence fertility [110]. 

A few studies have been performed to identify the eQTLs associated with fertility in cattle. Kadarmideen and Mazzoni [112] evaluated both donor and recipient dairy cows to determine the eQTLs modulating candidate genes that could predict in vitro produced and embryo transferred outcomes. These authors identified nine local eQTLs (*cis*-eQTLs) regulating *CCNB1* and *ROR1* genes in donor cows. Likewise, they reported two other candidate genes (*RARRES1* and *EMC9*) affected by 18 eQTLs [112]. These eQTLs were colocalized with previously reported QTLs for reproduction-related traits, suggesting that they may affect fertility through the regulation of gene expression. For example, the *CCNB1* and *ODF2L* genes were associated with average blastocyst rate, while *EMC9, MANBA*, and *PI16* were related to endometrial receptivity [112]. 

Using different approaches to identify eQTLs associated with fertility (measured as calving interval in days) in dairy cattle, van den Berg et al. [114] reported 89 QTLs and 554 genes with at least one eQTL variant within 1 Mb of the gene (*p*-value ≤ 10^−5^). Among the intervals containing eQTL and QTL for fertility, the genes *FAM184B, CDAN1*, and *SSTR1* were identified from white blood cells [114]. Forutan et al. [109] also conducted multiple analyses to identify 87 potential genes affecting four fertility traits in beef cattle based on the whole blood transcriptomes of pre-pubertal heifers and cows. According to the authors, five affected genes were over-represented in the extracellular matrix (ECM)-receptor interaction pathways. Interestingly, 8 out of the 87 genes were previously associated with age at natural menopause and age at menarche in humans [109]. As previously reported, a low level of overlapping between the GWAS and eQTL regions was observed (n = 8 genes). However, 11 regulatory variants within Chr 5 and 14 were confirmed in the validation population, supporting their inclusion in genotyping arrays for genomic evaluation. According to the authors, these variants were previously reported to harbor nearby genes associated with hormone production in Nelore cattle (*PLABG1* and *CHCHD7*) and oocyte competence (*ATP6V1C1*) [109].

## 4. Conclusions and Future Perspectives

This review showcases the advances that have resulted from implementing genomics and transcriptomics approaches to understand the genetic basis underlying fertility-related traits in cattle. Despite the progress made, genomic selection in beef cattle has been adopted much less than in dairy cattle [38]. Commercial tests are now available and have proven effective in increasing the accuracy of predictions for production traits [56]. However, their field performance for fertility-related traits is still lacking [41]. Due to their complex nature and low heritability, further studies on the impact and accuracy of commercial SNP arrays to select replacement females are needed. In this sense, most of the publications reviewed here have less relevance due to their limited sample sizes. Therefore, initiatives established by breeding associations to genotype and report the whole-herd phenotyping of animals are critical to design studies that provide more accurate EPDs. Likewise, accurate phenotyping for traditional fertility traits, as well as the development of novel traits, can improve genetic gain [33,37,40]. In this case, advances in high-throughput phenotyping technologies hold great potential to advance breeding programs. From a producer’s point of view, identifying early-in-life indicator traits for fertility can shorten generation intervals [33] and save on the resources invested in developing unproductive heifers. From a functional perspective, the complexity of the mechanisms and factors related to genome regulation reflects the challenge of linking the genome to the phenome. 

While we can profile the transcriptome, a comprehensive understanding of the effects of the up and downregulation of differentially expressed genes is still challenging. Better analytical methods are needed to go beyond gene lists and extract meaningful information for translatable applications [52,77]. Likewise, the crosstalk between immune systems and fertility is not fully understood, and further research is needed. Several studies have provided evidence to support the use of transcriptomics to identify predictive candidate biomarkers for female fertility. However, there is large transcriptional variation within and between tissues related to fertility genes in cattle [21,105]. As discussed, the limited overlapping of potential biomarkers across studies in the literature highlights the need for further research and the identification of non-invasive, tissue-specific candidate biomarkers. As for the GWAS studies, increased sample sizes and the validation of findings based on different cohorts are required to establish accuracy and consistency. 

The lack of existing research studies implementing multi-omics and multi-tissue analyses highlights the complexity of the mechanisms governing fertility. As more data from proteomics and metabolomics approaches become available, we can expect a better understanding of the genomic regulation of fertility traits. Similarly, multi-omics data integration will allow us to discover and prioritize candidate markers. Preliminary work from our group on machine learning and network modeling has provided a framework for prioritizing genes for fertility [28]. Approaches integrating transcriptomics into proteomics or metabolomics to identify candidates for early pregnancy diagnosis or fertility, respectively, have been described [81,115]. Additionally, eQTL analysis will enhance our understanding of the effects of genetic variants on gene expression and provide relevant SNP markers for trait-specific genomic selection [112]. Nonetheless, further research is needed to understand the molecular basis of gene expression variation [110]. Furthermore, new methods, including intermediate omics features, are being explored for genetic prediction [116]. Considering the complex nature of fertility, significant advances in precision breeding and the journey from bench to on-farm biomarker applications will rely on holistic approaches to further advance our ability to predict reproductive performance. 

## Figures and Tables

**Table 1:** The EPDs for beef cattle reproduction-related traits (reported by selected U.S. breed associations).

Trait (EPD Abbreviation)	Interpretation	Beef Cattle Breed Associations ^a^				
		AAA	AHA	RAA	ASA	AGA
Heifer pregnancy (HP/HPG ^b^)	Differences in the percentage of daughters who conceive and calve by 2 years of age.	×		×		×
Stayability (STAY)	Difference in daughters’ ability to remain in the herd and produce a calf through 6 years of age.			×	×	×
Sustained cow fertility (SCF)	Difference in cow’s ability to continue to calve from 3 to 12 years of age, given she calved as a 2-year-old.		×			
30-month pregnancy (Pg30)	Differences in the percentage of a sire’s daughters to conceive and calve at 3 years of age, given they calved as a first-calf heifer.					×
Scrotal circumference (SC) ^c^	Difference in the scrotal circumference of an animal’s male offspring compared to that of other sires.	×	×			×

^a^ AAA: American Angus Association; AHA: American Hereford Association; RAA: Red Angus Association of America; ASA: American Simmental Association; AGA: American Gelbvieh Association; ^b^ heifer pregnancy abbreviation used by RAA; ^c^ used as a proxy for female fertility and heifer age at puberty. Table based on Garrick [40].

**Table 2:** Commercially available genomic tests for fertility-related traits in beef cattle.

Traits	Test/Company ^a^	Breeds	Reference
Fertility	INHERIT Select^™^ Zoetis (Parsippany-Troy Hills, NJ, USA) ^c^	Angus, Red Angus, South Devon, Hereford, Simmental, Gelbvieh, Limousin and Charolais	[59]
Scrotal Circumference ^b^			
Heifer Pregnancy	GeneMax^®^ Advantage^™^ Zoetis ^c^	Angus (75% or greater)	[60]
Stayability	Igenity^®^+Envigor Neogen (Lansing, MI, USA)	Angus, Brahman, Gelbvieh, Hereford, Red Angus, Limousin, Simmental, and others	[61]
Heifer Pregnancy			
Heifer Pregnancy Rate	Igenity^®®^ Angus Gold Neogen	Angus (75% or greater)	[62]

^a^ Mention of a trade name or commercial product does not constitute or imply its endorsement by the authors; ^b^ used as a proxy for female fertility and heifer age at puberty; ^c^ exclusively for females.

## Data Availability

All relevant data are presented within the paper and the Supplementary Information files.

## Supplementary Materials

The following supporting information can be downloaded at: https://www.mdpi.com/article/10.3390/ani13203284/s1. Table S1: Summary of selected genome-wide association studies for fertility-related traits in beef cattle; Table S2: Summary of selected blood transcriptome studies for fertility-related traits in beef heifers.
